# First description of semiquantitative strain elastography in a dog with chronic inflammatory enteropathy

**DOI:** 10.29374/2527-2179.bjvm003725

**Published:** 2025-08-13

**Authors:** Iago Martins Oliveira, Rafaela Rodrigues Ribeiro, Daniel Vieira Costa, Wanessa Patrícia Rodrigues da Silva, Naida Cristina Borges

**Affiliations:** 1 Escola de Ciências Médicas e da Vida (ECMV), Pontifícia Universidade Católica de Goiás (PUC-GO). Campus II, Goiânia, GO, Brazil.; 2 Programa de Pós-Graduação em Ciência Animal (PPGCA), Escola de Veterinária e Zootecnia (EVZ), Universidade Federal de Goiás (UFG). Samambaia, Goiânia, GO, Brazil.; 3 Departamento de Medicina Veterinária (DMV), Escola de Veterinária e Zootecnia (EVZ), Universidade Federal de Goiás (UFG). Samambaia, Goiânia, GO, Brazil.

**Keywords:** canine, diagnostic imaging, inflammation, intestinal elasticity, canino, exame de imagem, inflamação, elasticidade intestinal

## Abstract

A 4-year-old female Shih Tzu presented with intermittent vomiting, diarrhea, and inappetence lasting 6 months. Physical examination revealed a mildly reduced body condition score (2/5), with no other significant abnormalities. Laboratory tests, imaging studies, and endoscopic evaluation confirmed the diagnosis of chronic inflammatory enteropathy. Strain elastography of the duodenum showed a semiquantitative strain ratio (SR) of 1.19 and a heterogeneous color pattern (blue–green with red areas), indicating increased tissue stiffness. Histopathological analysis revealed duodenitis and gastritis. This case highlights the potential of strain elastography as a non-invasive imaging tool for evaluating intestinal stiffness, correlating with inflammation, and supporting the diagnosis and management of canine enteropathies.

## Resumo

Uma cadela Shih-tzu de 4 anos de idade apresentou vómitos intermitentes, diarreia e inapetência durante seis meses. O exame físico revelou uma ligeira redução do escore de condição corporal (2/5), sem outras anormalidades. As análises laboratoriais, os exames imagiológicos e a endoscopia sugeriram enteropatia inflamatória crónica. A elastografia de tensão do duodeno revelou um strain ratio (SR) semiquantitativo de 1,19 e um padrão de cor heterogéneo (azul-verde com áreas vermelhas), indicando um aumento da rigidez dos tecidos. A histopatologia confirmou duodenite e gastrite. Este caso destaca o potencial da elastografia como uma ferramenta não invasiva para avaliar a rigidez intestinal, correlacionando-a com a inflamação e apoiando o diagnóstico e o tratamento de enteropatias caninas.

## Introduction

Elastography is an advanced imaging modality increasingly utilized in veterinary medicine for evaluating organs such as the thyroid gland, mammary tissue, and liver (Feliciano et al., 2023; Puccinelli et al., 2022; Ramos et al., 2024). However, limited data are available regarding its application for intestinal assessment in dogs. To date, only one study has explored shear wave elastography, establishing reference values for healthy jejunal mucosa and reporting findings in a case of histiocytic colitis (Cordella et al., 2021; Spużak et al., 2019). To the best of the authors’ knowledge, no studies have described the use of strain elastography for intestinal evaluation in dogs. Therefore, this pioneering case report aims to present the elastographic findings in a dog diagnosed with chronic inflammatory enteropathy.

## Case description

The patient was a 4-year-old female Shih Tzu dog weighing 7 kg who suffered from vomiting, diarrhea, and inappetence. During anamnesis, it was revealed that these clinical signs had appeared intermittently for 6 months. The physical examination revealed a slight decrease in the body condition score (2/5). The other parameters were consistent with the species’ reference values. The Chronic Enteropathy Activity Index was 9. Given this information, the presumptive diagnosis was alimentary enteropathy, either infectious, parasitic, or secondary to a chronic systemic disease.

A complete blood count, measurements of creatinine, alanine aminotransferase (ALT), alkaline phosphatase (ALP), albumin, total cholesterol, cobalamin, folate, C-reactive protein, basal bile acids, and basal cortisol as well as urinalysis, urinary protein-to-creatinine ratio, and trypsin-like immunoreactivity were performed. Additional diagnostic tests included an immunochromatographic assay for Giardia duodenalis, stool parasitology, and fecal RT-PCR to detect Cryptosporidium parvum, Giardia duodenalis, Clostridium perfringens, adenovirus, circovirus, parvovirus, and coronavirus. All test results were within normal limits. Detailed results are presented in Table 1. Abdominal ultrasonography revealed gastrointestinal wall thickening, with preservation of the normal layered pattern (stomach: 0.65 cm; duodenum: 0.40 cm; jejunum: 0.38 cm).

**Table 1:** Laboratory parameters of a dog with chronic inflammatory enteropathy.

**Complete blood count**		
	**Results**	**Reference range**
**Erythrogram**		
RBC	6.92 tera/L	5.50 – 8.50 tera/L
Hemoglobin	14.2 g/dL	12.0 – 18.0 g/dL
Hematocrit	40.0%	37.0 – 55.0%
MCV	68.5 fL	60.0 – 77.0 fL
MCHC	34.0%	32.0 – 36.0%
RDW	14.3%	-
Metarubricites	0/100	-
**Leucogram**		
WBC	9.800 /mm3	6.000 - 17.000 /mm3
Myelocytes	0	0
Metamielócitos	0	0
Neutrophils Rod	0 / 0	0 - 3 / 0 - 300
Segmented	69 / 6.893	60 - 77 / 3.000 - 11.500
Eosinophils	2 / 150	2 - 10 / 100 - 1.250
Basophils	0	0 / raros
Lymphocytes	15 / 2500	12 - 30 / 1.000 - 4.800
Monocytes	7 / 679	3 - 10 / 150 - 1.350
Platelets	350 mil/uL	200 - 500 mil/uL
TPP	7.4 g/dL	6.0 – 8.0 g/dL
**Biochemistry**		
Creatinine	1.1	0.50 – 1.50 mg/dL
ALT	60	21 – 102 UI/L
ALP	63	20 – 156 UI/L
Albumin	2.4	2,3 – 3,8 g/dL
Cholesterol	136	125 – 270 mg/dL
Cobalamin	430	252 – 908 pg/mL
Folate	5.8	3.5 – 8.5 ng/mL
C-reactive protein	10	< 20 mg/dL
Basal bile acids	7.8	0 – 14.9 mmol/L
Basal cortisol	1.5	0.5 – 5.5 mcg/dL
TLI	25	5.2 – 35 ng/mL

*Note*. RBCs: Red blood cell; MCV: Mean corpuscular volume; MCHC: Mean corpuscular hemoglobin concentration; RDW: Red cell distribution width; WBC: White blood cell; TPP: Total plasma protein; ALT: Alanine aminotransferase; ALP: Alkaline phosphatase; TLI: Trypsin-like immunoreactivity

**Figura 1:**
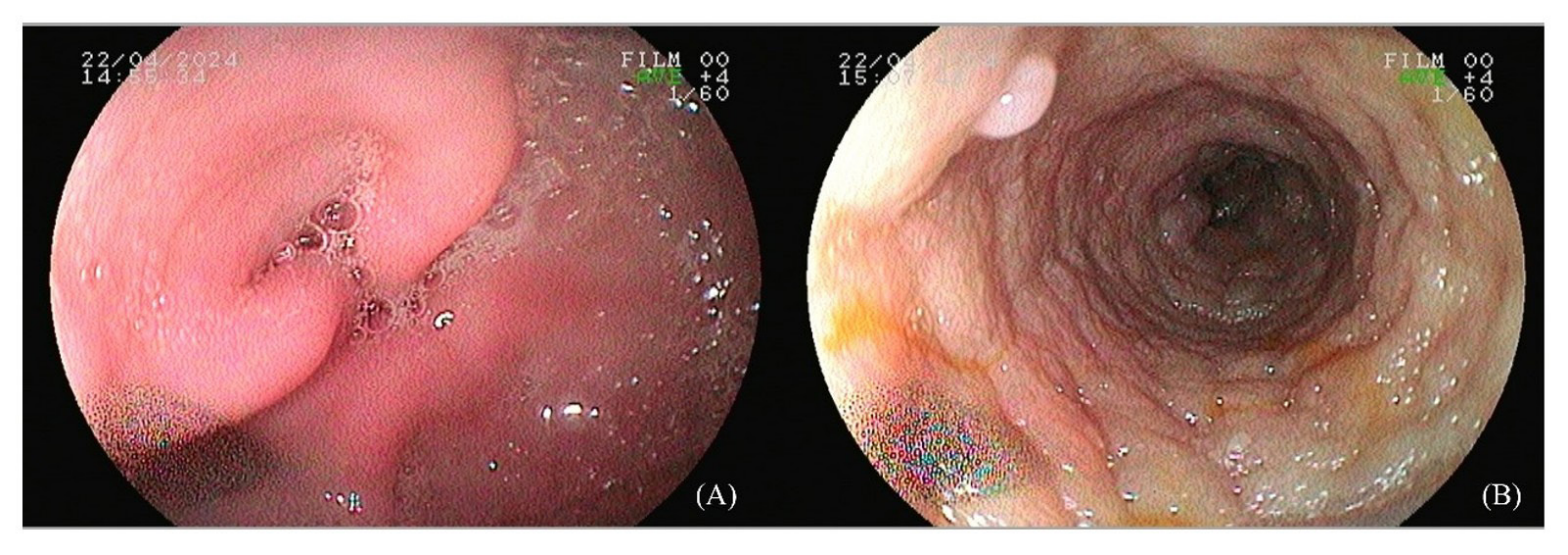
Upper digestive endoscopy of a dog with chronic inflammatory enteropathy. (A) Pyloric hyperplasia. (B) Edema of the duodenal mucosa associated with increased granularity and irregularity.

Based on the findings, a commercial symbiotic and hypoallergenic diet with hydrolyzed vegetable protein was prescribed for 30 days. The patient showed no clinical improvement, so an upper digestive endoscopy was performed, which revealed erythematous foci and pyloric hyperplasia (Figure 1A). The duodenum’s mucosae were irregular and swollen (Figure 1B). Biopsy samples were collected, and histopathological examination revealed hyperplastic gastritis, mild lymphoplasmocytic, and marked lymphoplasmocytic duodenitis with moderate fibrosis.

**Figura 2:**
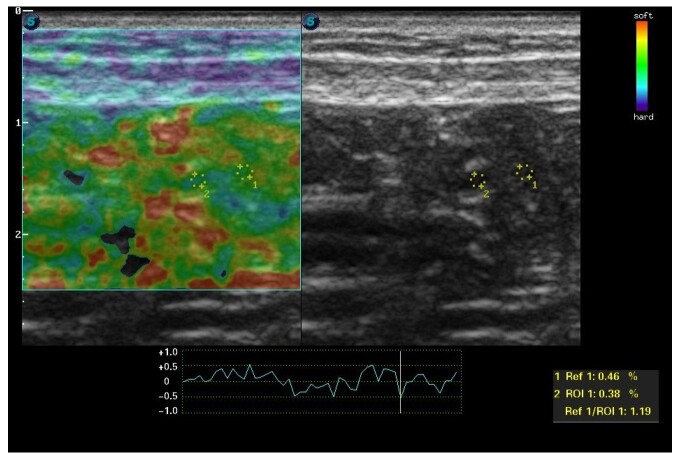
Elastography of duodenal mucosal deformation in a dog with chronic inflammatory enteropathy. Color elastogram and B-mode ultrasound image of the duodenum in cross-section.

*Note*. Note ROI 1 (yellow dashed circle) in the mesenteric region and ROI 2 (yellow dashed circle) in the dorsal mucosa of the duodenum. The elastographic stress curve and strain ratio can be seen at the bottom of the image.

**Figure 3:**
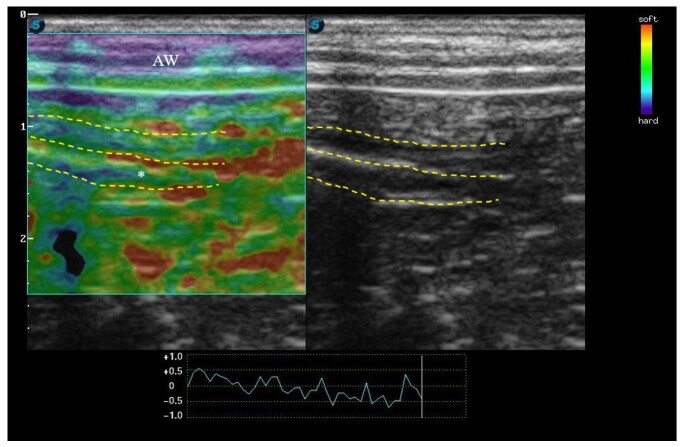
Dual-projection color elastogram with B-mode ultrasound image of the duodenum of a dog with chronic inflammatory enteropathy.

*Note*. The intestinal segment is delimited by yellow dashed lines. Longitudinal scan of the proximal duodenum with blue-green coloring (asterisk) in the mucosal region. Legend: AW - Abdominal wall.

Before treating chronic inflammatory enteropathy, elastography of the duodenum was used. The SAEVO FT422 equipment was used in conjunction with 8.0-MHz multifrequency linear transducer. Three cycles of abdominal compression and decompression were completed, followed by the creation of a color histogram with the image duplicated in B-mode. The color map ranged from blue to green and red. In the semiquantitative assessment, the proximal duodenum was evaluated in cross-section, and the reference region of interest (ROI) was drawn on the adjacent mesentery using the device's software, while ROI 2 was drawn on the dorsal part of the duodenal mucosa. The drawings were similar, circular, and had the same depth and horizontal direction. The SR was determined by calculating the ROI ratio. The ROI values for the duodenum were 0.46% and 0.38%, respectively, with an SR of 1.19 (Figure 2). For the static qualitative assessment, the duodenum was examined in a longitudinal ultrasound section, and a heterogeneous distribution was discovered between the dorsal and ventral regions of the intestine, with patterns ranging from predominantly blue to green with some reddish foci (Figure 3). Symbiotics, immunosuppressive drugs, and a highly digestible diet were used as treatment.

## Discussion

This note derived elastographic values and qualitative information on the elasticity of the duodenal mucosa in a dog with chronic inflammatory enteropathy. There are no directly comparable studies because this information has not been previously published. The results were obtained from the literature (Cordella et al., 2021; Spużak et al., 2019). However, it is important to note that the authors used the shear wave elastography technique in different intestinal segments than the one evaluated in the patient in this study.

The linear transducer had a frequency of 8 MHz because it was a small dog. This differs from the approach used in human intestinal assessment, which ranges between 3 and 11 MHz (Giannetti et al., 2019). This variation is similar to those used in hepatic, renal, jejunal, and orthopedic elastography in dogs (Puccinelli et al., 2022; Signore et al., 2021; Spużak et al., 2019; Tamura et al., 2019). Despite these similarities, we believe that the physical size of the animal, the depth of the organ under examination, and the type of elastography have a direct impact on the transducer and frequency used.

In humans, the intestinal color histogram is blue for stiffer tissues, green for intermediate hardness, and red for maximum elasticity (Giannetti et al., 2019). These results support the color map generated by elastography in this report. In the qualitative assessment of the patient described herein, the duodenal mucosa was predominantly blue–green with reddish foci, indicating increased intestinal hardness. There is no colorimetric scoring system in veterinary medicine for interpreting the distribution of colors in intestinal elastography; however, the findings are classified as inflamed intestine (blue–green) based on a pediatric score that also determined the color pattern for healthy intestine (red–green) and fibrosis (blue) (Fufezan et al., 2015).

In human medicine, transverse regions of interest are defined between the muscle wall and the intestinal mucosa (Gabbiadini et al., 2021; Quaia et al., 2018). In contrast to what we did in this case, where intestinal hardness was compared longitudinally to the adjacent mesentery, the goal was to evaluate tissues of equal depth. In addition, we did not use the muscle wall as a reference tissue because its components are stiffer than those of the intestine.

The duodenal SR in the case described was 1.19, which is consistent with the literature, which states that SRs >1 indicate greater hardness of the tissue being assessed (Choi et al., 2015). Despite this, this semiquantitative reference is nonspecific because it applies to multiple organs and was obtained through lymphatic evaluation. We propose that future studies establish standardized values for dog intestines.

## Conclusion

Strain elastography is an advanced, non-invasive diagnostic imaging modality that assesses tissue hardness and, when applied to the gastrointestinal tract, can suggest tissue fibrosis, which may have an impact on animal prognosis by categorizing the severity of the intestinal disease as well as attempting to verify the degree of evolution of the inflammatory condition and the response to therapy. In this case, the increased intestinal hardness seen on elastography corresponded to the histopathological description of the inflammatory condition. However, serial follow-up could help to better understand the potential of elastography in intestinal assessment. This was not possible in this case and represents a limitation. Finally, we propose that this study be used in future research to investigate advanced techniques for diagnosing canine enteropathies.
